# MRPL37 promotes hepatocellular carcinoma progression through modulating mitochondrial energy metabolism

**DOI:** 10.1016/j.isci.2025.114052

**Published:** 2025-11-14

**Authors:** Yigan Zhang, Minjie Chen, Huidi Li, Hao Deng, Shuwen Chen, Jiaxin Ni, Junjie Hu, Sixian Lei, Linsheng Huang, Shuangsuo Dang, Zhuoshun Yang, Wuhua Zhou, Deping Ding, Yanbin Dong, Zhongji Meng

**Affiliations:** 1 Institute of Biomedical Research, Department of Infectious Diseases, Regulatory Mechanism and Targeted Therapy for Liver Cancer Shiyan Key Laboratory, Hubei Provincial Clinical Research Center for Precise Diagnosis and Treatment of Liver Cancer, Taihe Hospital (First Clinical College of Medicine), Hubei University of Medicine, Shiyan, Hubei 442000, China; 2 Department of Pathology, The Affiliated Lianyungang Hospital of Xuzhou Medical University, Lianyungang Clinical College of Nanjing Medical University, The First People’s Hospital of Lianyungang, Lianyungang 222061, China; 3 Department of Hepatobiliary Pancreatic Surgery, Affiliated Taihe Hospital, Hubei University of Medicine, Shiyan, Hubei, China; 4 Hubei Key Laboratory of Tumor Microenvironment and Immunotherapy, College of Basic Medical Science, China Three Gorges University, Yichang 443002, China; 5 Department of Infectious Diseases, The Second Affiliated Hospital of Xi’an Jiaotong University, Xi’an 710004, China

**Keywords:** human metabolism, organizational aspects of cell biology, cancer

## Abstract

Mitochondrial ribosomal proteins of the large subunit (MRPLs) are critical for mitochondrial function and cellular energy metabolism. However, the role of the MRPL family in hepatocellular carcinoma (HCC) remains poorly understood. Here, we leveraged The Cancer Genome Atlas (TCGA) liver cancer data to develop a subtype classification and prognostic model based on the MRPL family genes, identifying MRPL37 as a key gene associated with HCC progression. Clinically, MRPL37 upregulation is associated with HCC progression and poor prognosis. Functionally, MRPL37 knockdown significantly inhibits HCC cell proliferation, disrupts cell cycle progression, and induces apoptosis *in vitro*. *In vivo*, silencing MRPL37 reduces tumor growth in both xenograft and spontaneous liver cancer models. Mechanistically, MRPL37 regulates mitochondrial protein synthesis, influencing key metabolic pathways and mitochondrial function, including oxidative phosphorylation. Our results suggest MRPL37 as a critical regulator of energy metabolism in HCC, highlighting its potential as a therapeutic target for liver cancer.

## Highlights

• Developed a subtype classification and prognostic model for HCC based on MRPL family genes
• MRPL37 upregulation is correlated with poor prognosis in HCC
• MRPL37 knockdown inhibits HCC cell proliferation both *in vitro* and *in vivo*
• MRPL37 promotes HCC progression by regulating mitochondrial energy metabolism

## Graphical abstract

**Figure:**
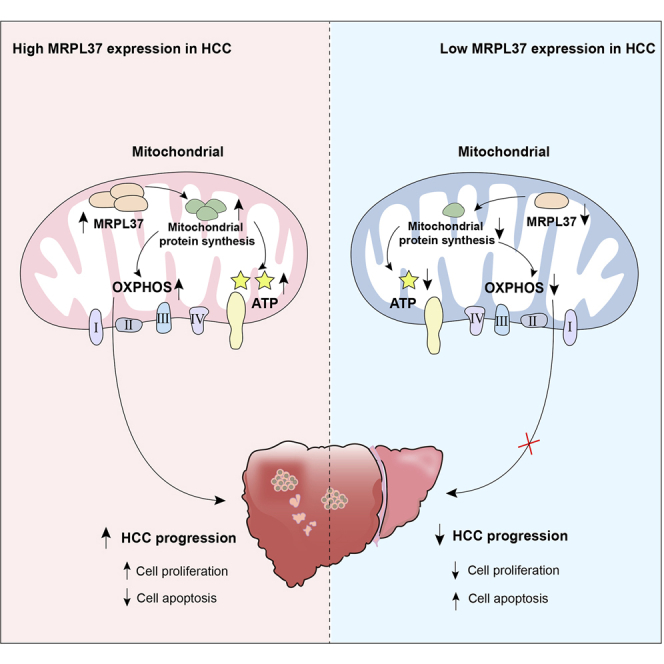

## Introduction

Hepatocellular carcinoma (HCC) is one of the most prevalent and aggressive malignancies globally, with particularly high incidence and mortality rates in China.^1^ According to the National Health Commission and the National Cancer Center, liver cancer is the fourth most common cancer in China and the second leading cause of cancer-related deaths, with over 380,000 new cases and more than 330,000 deaths annually.^2^ Despite recent advancements in clinical treatments, including surgery, liver transplantation, targeted therapies, and immunotherapy, the overall therapeutic efficacy remains suboptimal due to challenges in early diagnosis, treatment resistance, and the disease’s complex biological characteristics. The mortality rate for liver cancer remains high, with a survival rate of only around 20%.^3^^,^^4^^,^^5^ Thus, there is an urgent need to explore new molecular mechanisms underlying HCC development to inform the development of novel targeted therapies.

Energy metabolism reprogramming is a hallmark of cancer cells, with altered metabolic pathways supporting the rapid growth and survival of tumors.^6^^,^^7^ Mitochondria, as the central organelles responsible for energy metabolism, play a crucial role in this process, particularly through oxidative phosphorylation (OXPHOS). Mitochondrial dysfunction has been recognized as a key driver of various cancers, including HCC.^8^^,^^9^ For example, mitochondrial dysfunction can lead to reactive oxygen species (ROS) production, causing DNA damage and genomic instability, which accelerates cancer progression.^10^ Investigating mitochondrial metabolism abnormalities in HCC provides valuable insights into tumorigenesis and highlights potential therapeutic targets.

The mitochondrial ribosomal proteins of the large subunit (MRPLs) are essential components of the mitochondrial ribosome, involved in mitochondrial protein synthesis.^11^ Recent studies have shown that MRPL family members are implicated in various cancers.^12^ For example, MRPL44 is a predictor of lymph node metastasis in papillary thyroid carcinoma.^13^ In liver cancer, MRPL13 inhibition has been identified as a key regulator of OXPHOS dysfunction and increased invasiveness.^14^ However, MRPL13 acts as an oncogene in lung adenocarcinoma and breast cancer.^15^^,^^16^ Additionally, dysregulation of MRPL expression promotes an aggressive HCC phenotype by inhibiting immune surveillance.^17^ These findings suggest that MRPL dysregulation is a primary cause of mitochondrial metabolic disruptions, contributing to cancer progression. However, the role of MRPLs in liver cancer remains poorly understood, and their underlying mechanisms have not been fully elucidated.

This study aims to develop a novel liver cancer subtype and prognostic model based on MRPL family gene expression using The Cancer Genome Atlas (TCGA) liver cancer cohort RNA sequencing (RNA-seq) data. Among MRPL genes, MRPL37 was identified as a key gene with significant prognostic value. We further investigated the functional role of MRPL37 in liver cancer progression through *in vitro* and *in vivo* models, demonstrating its regulatory impact on mitochondrial metabolism and tumorigenesis. These findings suggest that MRPL37 could be a promising therapeutic target for liver cancer treatment.

## Results

### Construction of a subtype classification model based on MRPL family genes

**Figure 1:**
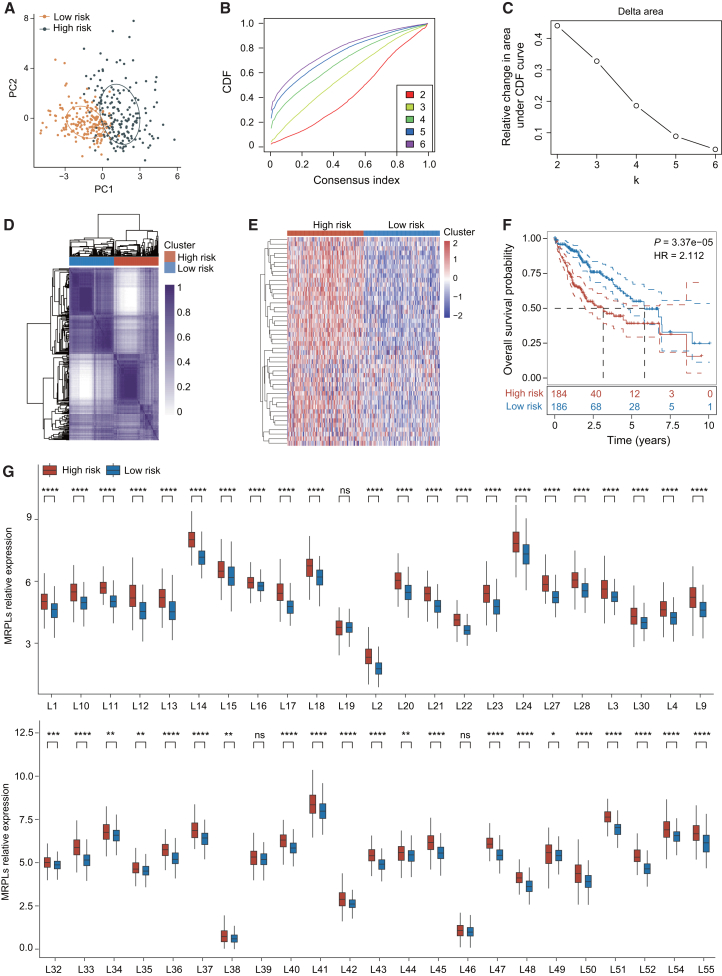
MRPL family-based liver cancer classification model and survival analysis (A) Principal component analysis (PCA) showing distinct gene expression patterns between the high-risk and low-risk groups based on MRPL family gene expression. (B) Consensus cumulative distribution function (CDF) curve used to determine the optimal number of clusters for stratifying liver cancer samples into high-risk and low-risk groups. (C) Delta area analysis confirming the two-cluster model as the most appropriate for classification. (D) Clustering heatmap illustrating the expression differences of MRPL family genes between the high-risk and low-risk groups. (E) Heatmap analysis revealing differential expression of MRPL family genes across the two groups. (F) Kaplan-Meier survival curve showing significantly poorer overall survival in the high-risk group compared to the low-risk group. (G) Expression analysis of MRPL family genes in the high-risk and low-risk group. Data in G are presented as the mean ± SD. ∗*p* < 0.05, ∗∗*p* < 0.01, ∗∗∗*p* < 0.001, ∗∗∗∗*p* < 0.0001. ns, not significant. Two-tailed Student’s *t* test.

To identify MRPL family gene expression-based subtypes in liver cancer, we analyzed RNA-seq data and clinical information from 371 HCC samples in the TCGA database. Consistent clustering analysis was employed to classify the samples into high-risk (*n* = 185) and low-risk (*n* = 186) groups based on MRPL family expression patterns. Principal component analysis revealed a clear distinction between these two groups in terms of gene-expression patterns (Figure 1A). The optimal number of clusters was determined using the consensus cumulative distribution function and delta area analysis (Figures 1B and 1C), with the results suggesting that a two-cluster model best separated the samples into the high- and low-risk categories. A clustering heatmap further confirmed the separation between the two groups, highlighting distinct differences in MRPL family gene expression (Figures 1D and 1E). Survival analysis demonstrated that the high-risk group had significantly poorer overall survival compared to the low-risk group (Figure 1F). Additionally, gene-expression analysis of the MRPL family revealed that in the high-risk group, 44 genes were significantly upregulated, while only two genes showed no significant difference in expression (Figure 1G).

Subsequently, differential gene expression analysis was performed between the high-risk and low-risk groups based on RNA-seq data from the TCGA database. Volcano plot and heatmap analysis revealed that 66 genes were upregulated and 90 genes were downregulated (Figures S1A and S1B). Gene ontology (GO) and Kyoto Encyclopedia of Genes and Genomes (KEGG) enrichment analyses indicated that the upregulated genes were primarily enriched in pathways promoting tumor proliferation, including cell division, cell cycle, and ribosome activity, while downregulated genes were predominantly involved in metabolic pathways such as fatty acid metabolism and glycolysis/gluconeogenesis (Figures S1C–S1F). These findings suggest that the high-risk group exhibits significant alterations in energy metabolism and growth regulation. Additionally, the one-class logistic regression algorithm was used to assess tumor stemness characteristics, revealing that the high-risk group had significantly higher tumor stemness scores than the low-risk group, indicating a more malignant phenotype (Figure S1G). Immune cell infiltration analysis using CIBERSORT further revealed that naive B cell infiltration was significantly reduced in the high-risk group, while regulatory T cell infiltration and activated NK cell were markedly increased (Figure S1H). Moreover, analysis of immune checkpoint gene expression showed that immune evasion genes such as CTLA4 and PDCD1 were significantly upregulated in the high-risk group (Figure S1I), suggesting that these tumors have a greater capacity for immune escape. Tracking of indels by decomposition (TIDE) analysis demonstrated that the high-risk group had a higher TIDE score compared to the low-risk group, indicating a poorer response to immune checkpoint inhibitors (Figure S1J).

### Construction of a prognostic model based on MRPL family genes

**Figure 2:**
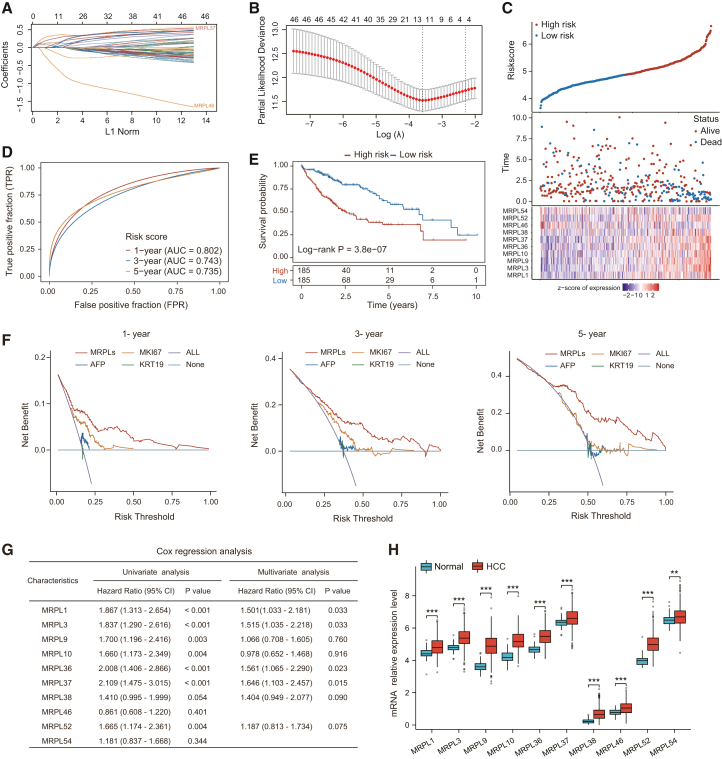
Prognostic model based on MRPL gene family and its validation (A and B) Construction of the MRPL gene prognostic model using LASSO regression and identification of key genes for risk score calculation. (C) Stratification of liver cancer patients into high-risk and low-risk groups based on the risk scores derived from the model, along with a heatmap showing the expression of MRPLs genes in relation to patient survival status. (D) Time-dependent ROC curves demonstrating the accuracy of the risk model in predicting 1-, 3-, and 5-year survival in liver cancer patients. (E) Kaplan-Meier survival analysis revealing significant survival differences between high-risk and low-risk groups. (F) DCA decision curve analysis comparing the MRPLs-based prognostic model with traditional tumor biomarker models (AFP, MKI67, and KRT19) for clinical prediction accuracy at 1-, 3-, and 5-year survival. (G) Univariate and multivariate Cox analyses showing the prognostic value of 10 candidate genes related to MRPLs. (H) mRNA expression levels of MRPLs-related 10 candidate genes in HCC tissues (*n* = 371) compared to normal tissues (*n* = 50). Data in H are presented as the mean ± SD. ∗∗*p* < 0.01, ∗∗∗*p* < 0.001. ns, not significant. Two-tailed Student’s *t* test.

To develop a novel prognostic model for liver cancer based on the MRPL family, we performed least absolute shrinkage and selection operator (LASSO) regression analysis using the TCGA HCC cohort. The results revealed that we identified 10 candidate genes for the risk score model (Figures 2A and 2B), and the calculation formula is as follows: risk score = (0.1797∗MRPL1) + (0.3634∗MRPL3) + (0.127∗MRPL9) + (0.0444∗MRPL10) + (0.1926∗MRPL36) + (0.3008∗MRPL37) + (0.1704∗MRPL38) + (−0.7307∗MRPL46) + (0.0688∗MRPL52) + (−0.2239∗MRPL54). Using the calculated risk scores, liver cancer patients were classified into high-risk and low-risk groups. With the increase in risk score, the overall survival of liver cancer patients gradually decreased and the deaths steadily increased (Figure 2C). Moreover, area under curve (AUC) values for 1-, 3-, and 5-year survival based on this risk model were 0.802, 0.743, and 0.735, respectively, indicating that the model performed well in predicting long-term survival (Figure 2D). Survival analysis revealed that liver cancer samples in the low-risk cluster had a longer survival compared to the high-risk group (Figure 2E). To further validate the clinical utility of the model, we constructed a decision curve analysis (DCA) to compare the prognostic performance of the MRPL family model with that of other established tumor biomarkers, including AFP, MKI67, and KRT19. The results demonstrated that the MRPL family-based DCA model provided superior clinical prognostic prediction compared to the other models (Figure 2F). Additionally, we further performed univariate and multivariate Cox analyses on the ten candidate genes and examined their mRNA expression in liver cancer tissues. The results showed that MRPL37 had the highest hazard ratio, and its mRNA level was also significantly upregulated in liver cancer tissues (Figures 2G and 2H). Therefore, we focused on MRPL37 for further investigation of its function and mechanisms in HCC.

### MRPL37 upregulation correlates with HCC progression and poor prognosis

**Figure 3:**
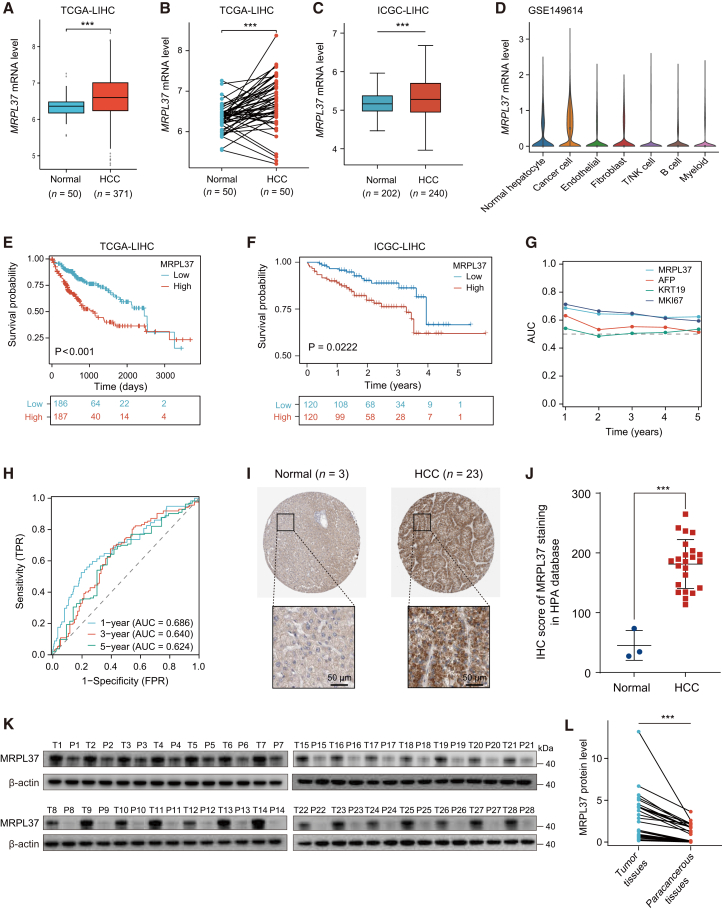
MRPL37 upregulation in HCC and its association with patient prognosis (A–C) Boxplot showing MRPL37 mRNA expression between normal and HCC tissues in the TCGA-LIHC cohort and ICGC-LIHC cohort. (D) Violin plot showing MRPL37 mRNA expression across different cell types in the GSE149614 dataset. (E and F) Kaplan-Meier survival curves based on MRPL37 expression in the TCGA-LIHC and ICGC-LIHC cohorts. (G) Time-dependent ROC curves assessing the predictive accuracy of MRPL37 for 1-, 3-, and 5-year survival in HCC. (H) ROC curves evaluating the sensitivity and specificity of MRPL37 at different time points. (I) Representative IHC staining of MRPL37 in normal and HCC tissues from HPA database (scale bars represent 50 μm). (J) Quantification of MRPL37 IHC staining in normal and HCC tissues. (K and L) WB analysis of MRPL37 protein expression in HCC tissues and adjacent tissues (*n* = 28, *n* represents number of patients). Data in J and L are presented as the mean ± SD. ∗∗∗*p* < 0.001. Two-tailed Student’s *t* test.

To investigate the role of MRPL37 in HCC, we first analyzed its expression in HCC samples from both the TCGA and International Cancer Genome Consortium databases. The results demonstrated that MRPL37 expression was significantly elevated in HCC tissues compared to normal liver tissues in both databases (Figures 3A and 3B). Additionally, single-cell RNA-seq data from liver cancer tissues (GSE149614 and GSE166635) showed that MRPL37 was highly expressed in cancer cells compared to other cell types (Figures 3C and S2A). Kaplan-Meier survival analysis further confirmed that patients with higher MRPL37 expression exhibited significantly worse overall survival (Figures 3D and 3E). Time-dependent AUC analysis revealed that the prognostic model based on MRPL37 showed better predictive ability compared to traditional markers like AFP, KRT19, and MKI67, with AUC values of 0.686 for 1 year, 0.640 for 3 years, and 0.624 for 5 years, suggesting MRPL37’s strong prognostic value (Figures 3F and 3G). Immunohistochemical (IHC) analysis of HCC tissue microarrays from the Human Protein Atlas database revealed that MRPL37 expression was significantly upregulated in HCC tissues compared to normal liver tissues (Figures 3H and 3I). Similarly, western blot (WB) analysis of fresh tumor tissues from 28 HCC patients confirmed the upregulation of MRPL37 expression in tumor tissues compared to adjacent non-cancerous tissues (Figures 3J and 3K). Furthermore, we conducted an analysis to investigate the correlation between MRPL37 expression and various clinical pathological factors, including age, gender, pathological stage, and pathological TNM stage. Our findings reveal a significant association between elevated MRPL37 expression and an advanced pathological T stage, suggesting that higher MRPL37 expression is correlated with greater malignancy and progression of HCC (Table S2).

Further, tumor stemness analysis showed that the mRNAsi score was significantly higher in the MRPL37 high-expression group, suggesting its association with enhanced tumor stemness and resistance to chemotherapy and radiotherapy (Figure S2B). In immune analysis, CIBERSORT results indicated that the infiltration of naive B cells and CD4^+^ memory T cells was significantly reduced in the MRPL37 high-expression group, while the infiltration of M2 macrophages was significantly increased (Figure S2C). Additionally, immune checkpoint gene analysis revealed that immune evasion genes such as CD274 (PD-L1) and HAVCR2 (TIM-3) were significantly upregulated in the MRPL37 high-expression group (Figure S2D). TIDE analysis further indicated that high-expression patients had higher TIDE scores, suggesting poor response to immune checkpoint inhibitors and enhanced immune evasion (Figure S2E). GO analysis revealed that positively correlated genes were enriched in pathways promoting tumor proliferation, such as nuclear division and DNA replication, while negatively correlated genes were associated with energy metabolism pathways, including amino acid and lipid metabolism (Figures S2F and S2G). KEGG pathway analysis revealed that positively correlated genes were mainly involved in cancer-related pathways, such as cell cycle, DNA replication, and p53 signaling pathway, while negatively correlated genes were enriched in metabolism pathways such as histidine metabolism, glycolysis, and gluconeogenesis (Figures S2H and S2I). Collectively, these findings suggest that MRPL37 upregulation correlates closely with malignant HCC progression and poor prognosis.

### MRPL37 facilitates tumorigenic potentials of liver cancer *in vitro and in vivo*

**Figure 4:**
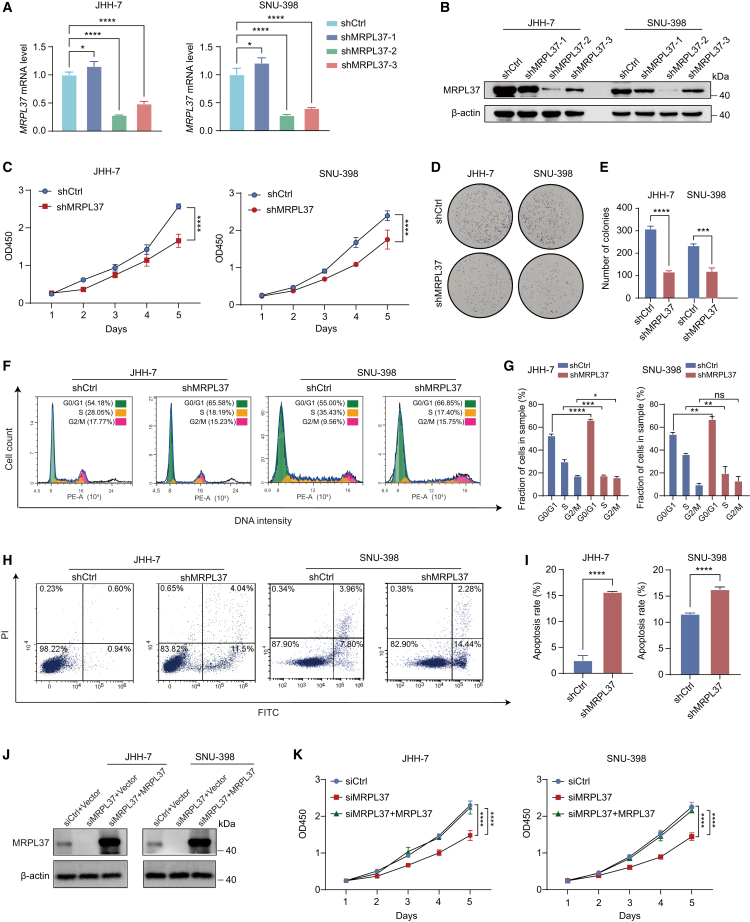
MRPL37 knockdown inhibits HCC cell proliferation, cell cycle, and induces apoptosis *in vitro* (A and B) qPCR and WB analysis showing the mRNA and protein levels of MRPL37 in JHH-7 and SNU-398 cells upon MRPL37 knockdown. (C) CCK-8 assay showing the effect of MRPL37 knockdown on cell proliferation in JHH-7 and SNU-398. (D and E) Colony formation assay showing the effect of MRPL37 knockdown on cell proliferation in JHH-7 and SNU-398. (F and G) Flow cytometry analysis of the cell cycle distribution in JHH-7 and SNU-398 cells after MRPL37 knockdown. (H and I) Flow cytometry analysis showing cell apoptosis in JHH-7 and SNU-398 cells after MRPL37 knockdown cells. (J) WB validates the restored MRPL37 expression upon MRPL37 knockdown in in JHH-7 and SNU-398 cells. (K) CCK-8 assay shows the effect of restored MRPL37 expression on cell proliferation upon MRPL37 knockdown in in JHH-7 and SNU-398 cells. Data in A, C, E, G, I, and K are presented as the mean ± SD (*n* = 3, n represents number of biological replicates). ∗*p* < 0.05, ∗∗*p* < 0.01, ∗∗∗*p* < 0.001. ∗∗∗∗*p* < 0.0001, ns, not significant. Two-tailed Student’s *t* test, One-way ANOVA with Tukey’s test and two-way ANOVA with post-hoc tests.

To investigate whether MRPL37 exerts oncogenic roles in HCC cells, we first performed MRPL37 knockdown in HCC cell lines and assessed its impact on cell phenotypes. Among seven commonly used HCC cell lines, qPCR and WB analyses revealed higher MRPL37 expression in JHH-7 and SNU-398 cell lines, which were selected for further phenotypic validation (Figures S3A and S3B). We chose the shRNA targeting MRPL37 with the highest knockdown efficiency for subsequent experiments (Figures 4A and 4B). CCK-8 cell proliferation and colony formation assays showed that MRPL37 knockdown significantly inhibited HCC cell proliferation (Figures 4C–4E). Furthermore, cell cycle analysis demonstrated that silencing MRPL37 induced G0/G1 phase cell-cycle arrest, suggesting that MRPL37 plays a key role in regulating cell cycle progression (Figures 4F and 4G). Additionally, apoptosis assays revealed that MRPL37 knockdown promoted cell apoptosis, further indicating its role in HCC cell survival (Figures 4H and 4I). To further validate the key role of MRPL37 in HCC cells, we performed MRPL37 rescue experiments. The results demonstrated that overexpression of MRPL37 in HCC cells with MRPL37 knockdown significantly rescued the impaired cell proliferation induced by MRPL37 knockdown (Figures 4J and 4K).

**Figure 5:**
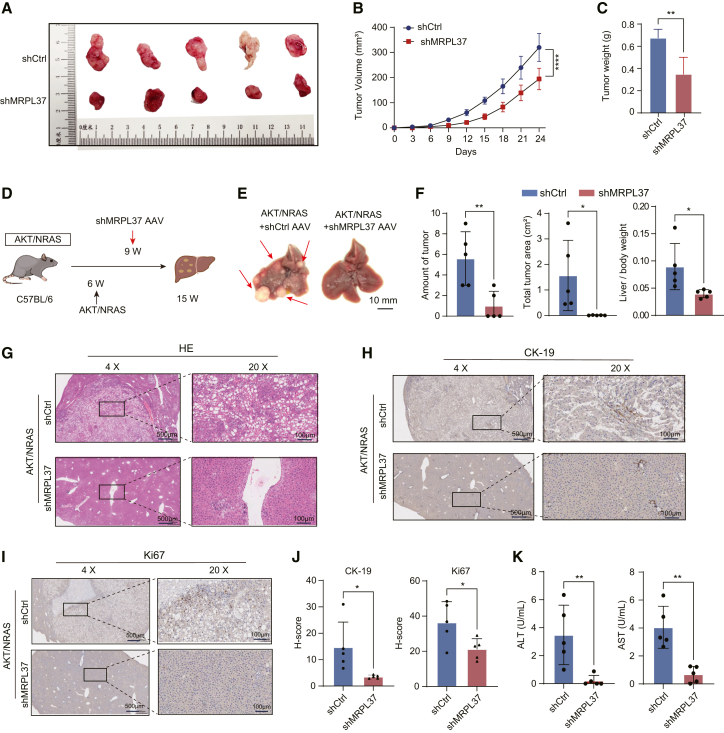
MRPL37 knockdown attenuates tumorigenesis of liver cancer *in vivo* (A) Representative images of tumors derived from mice with MRPL37 knockdown and control groups in the HCC xenograft model (*n* = 5, *n* represents number of mice). (B and C) Tumor volume growth curves and tumor weight analysis comparing the MRPL37 knockdown and control groups. (D) Schematic diagram of the AKT/NRAS oncogene-driven liver cancer model used to evaluate the impact of MRPL37 knockdown on hepatocarcinogenesis in C57BL/6 mice. (E) Representative images of liver tumors in the AKT/NRAS model showing the effect of MRPL37 knockdown on tumor formation and growth (scale bars represent 10 mm). (F) Quantification of amount of tumor (left), total tumor area (middle), and the liver-to-body weight ratio (right) in the AKT/NRAS model following MRPL37 knockdown. (G) Representative H&E staining images of liver tissues from the AKT/NRAS model after MRPL37 knockdown (left, scale bars represent 500 μm; right, scale bars represent 100 μm). (H and I) Representative IHC images for CK-19 and Ki67 in liver tissues from the AKT/NRAS model following MRPL37 knockdown (left, scale bars represent 500 μm; right, scale bars represent 100 μm). (J and K) Quantification of IHC results for CK-19, Ki67, and serum levels of AST and ALT in the AKT/NRAS model following MRPL37 knockdown. Data in B, C, F, J, and K are presented as the mean ± SD (*n* = 5, n represents number of mice). ∗*p* < 0.05, ∗∗*p* < 0.01. ∗∗∗∗*p* < 0.0001. Two-tailed Student’s *t* test, One-way ANOVA with Tukey’s test and two-way ANOVA with post-hoc tests.

To assess the effect of MRPL37 on tumorigenicity *in vivo*, we performed tumor xenograft studies. The results showed that MRPL37 silencing significantly inhibited tumor growth, with marked reductions in both tumor volume and weight, compared to the control group (Figures 5A–5C). Moreover, we examined the impact of MRPL37 on hepatocarcinogenesis using the *AKT-NRAS* oncogene-driven model in C57BL/6 mice (Figure 5D). In the spontaneous liver cancer model, MRPL37 knockdown significantly impaired liver tumor formation, as evidenced by fewer and smaller tumors compared to the control group (Figures 5E and 5F). Histological analysis through hematoxylin and eosin staining revealed reduced the tumor burden in the MRPL37 knockdown group (Figure 5G). IHC analysis further demonstrated significantly lower expression levels of tumor biomarkers CK-19 and Ki67 in liver samples from the MRPL37 knockdown group (Figures 5H–5J). Moreover, silencing MRPL37 resulted in significant improvement in liver function, as indicated by reduced serum alanine transaminase and aspartate transaminase levels in the MRPL37 knockdown group (Figure 5K). Collectively, these findings suggest that MRPL37 plays an essential role as an oncogenic driver in liver cancer tumorigenicity both *in vitro and in vivo*.

### MRPL37 is closely correlated with oxidative phosphorylation and protein synthesis in HCC cells

**Figure 6:**
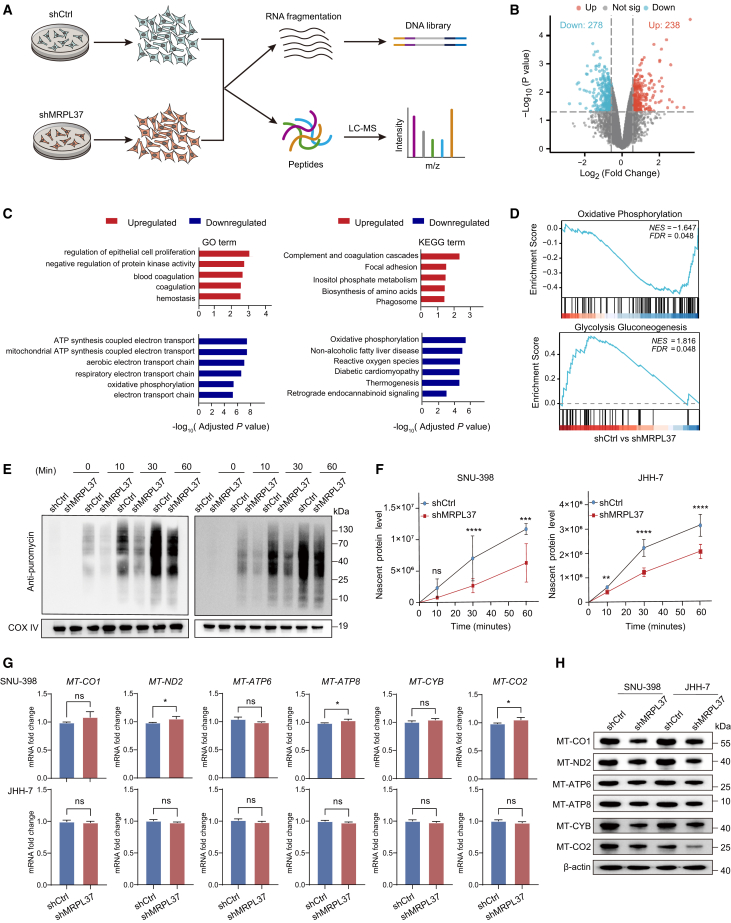
MRPL37 is closely associated with oxidative phosphorylation and protein synthesis in HCC cells (A) Workflow diagram showing the extraction of total RNA and proteins from the shCtrl and shMRPL37 groups in SNU-398 cells for transcriptomics and proteomics analysis. (B) Volcano plot displaying differentially expressed proteins (DEPs) between the shCtrl and shMRPL37 groups. (C) GO and KEGG pathway enrichment analysis of MRPL37-related DEPs. (D) GSEA pathway enrichment analysis of MRPL37-related DEPs. (E and F) WB analysis and quantification of nascent protein synthesis following MRPL37 knockdown at different time points in SNU-398 and JHH-7 cells. (G and H) qPCR and WB showing mRNA and protein levels of mitochondrial genes (MT-CO1, MT-ND2, MT-ATP6, MT-ATP8, MT-CYB, MT-CO2) in SNU-398 and JHH-7 cells following MRPL37 knockdown. Data in F and G are presented as the mean ± SD (*n* = 3, n represents number of biological replicates). ∗*p* < 0.05, ∗∗∗*p* < 0.001. ∗∗∗∗*p* < 0.0001. ns, not significant. Two-tailed Student’s *t* test, One-way ANOVA with Tukey’s test and two-way ANOVA with post-hoc tests.

To explore the mechanism through which MRPL37 contributes to tumorigenicity in HCC cells, we performed transcriptomic and proteomic analyses in SNU-398 cells after MRPL37 knockdown (Figure 6A). First, RNA-seq and differential expression gene (DEG) analysis revealed 83 upregulated and 130 downregulated MRPL37-related DEGs (Figure S4A). This result suggests that MRPL37 knockdown does not cause significant changes at the transcriptional level. Next, proteomic analysis identified 238 upregulated and 278 downregulated differentially expressed proteins (DEPs) associated with MRPL37 (Figure 6B). GO and KEGG pathway analyses showed that the downregulated DEPs were mainly enriched in biological processes related to OXPHOS, ATP synthesis coupled with electron transport, and other mitochondrial functions (Figure 6C). Additionally, Gene Set Enrichment Analysis indicated that MRPL37 was significantly associated with pathways related to OXPHOS and glycolysis gluconeogenesis, further highlighting its role in energy metabolism regulation (Figure 6D).

To understand how MRPL37 might influence mitochondrial functions, we used puromycin labeling to measure nascent protein levels and assess the impact of MRPL37 on protein synthesis. The results showed that MRPL37 knockdown in both SNU-398 and JHH-7 cells significantly reduced the levels of nascent proteins (Figures 6E and 6F). To further validate MRPL37’s involvement in mitochondrial energy metabolism, we examined the expression levels of six key mitochondrial energy metabolism proteins—MT-CO1, MT-ND2, MT-ATP6, MT-ATP8, MT-CYB, and MT-CO2—using qPCR and WB analysis (Figures 6G and 6H). As shown in the heatmap (Figure S4B), MRPL37 knockdown did not significantly affect the mRNA levels of these mitochondrial targets; however, the protein levels of these mitochondrial proteins were notably decreased in both SNU-398 and JHH-7 cell lines. These findings suggest that MRPL37 regulates energy metabolism in liver cancer cells by influencing the expression of key mitochondrial proteins, which play crucial roles in OXPHOS.

### MRPL37 knockdown affects mitochondrial function in HCC cells

**Figure 7:**
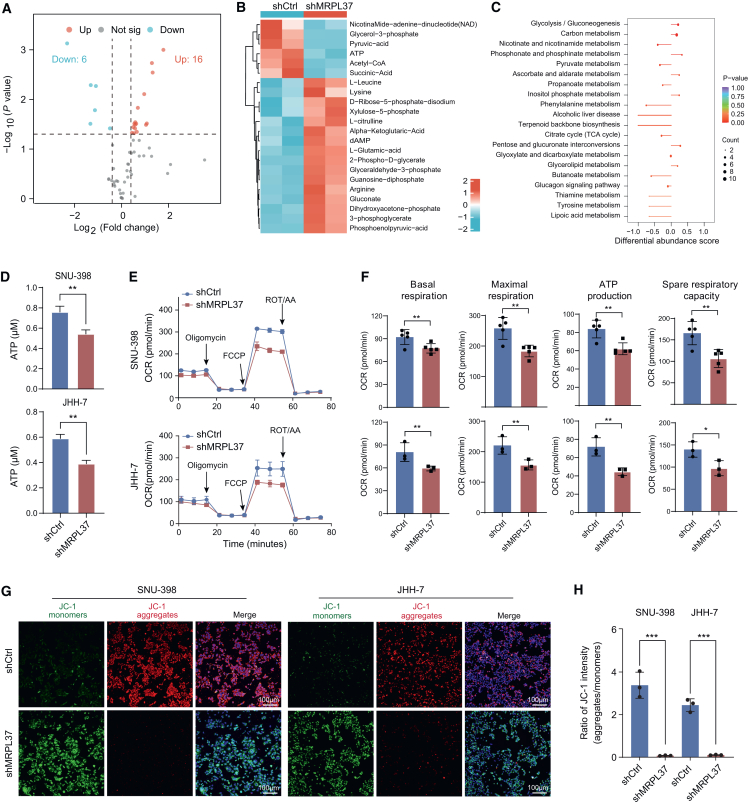
MRPL37 knockdown affects mitochondrial function in HCC cells (A) Volcano plot showing differential metabolomic products between the shCtrl and shMRPL37 groups. (B) Heatmap analysis of metabolites displaying differential abundance between shCtrl and shMRPL37 groups. (C) Differential abundance analysis revealing enriched signaling pathways associated with MRPL37 knockdown. (D) ATP content measurement analysis of ATP levels in both SNU-398 and JHH-7 cells following MRPL37 knockdown (*n* = 3, n represents number of biological replicates). (E) OCR measurements over time in SNU-398 and JHH-7 cells following MRPL37 knockdown. (F) Quantification of basal respiration, maximal respiration, ATP production, and spare respiratory capacity based on OCR measurements in SNU-398 (upper, *n* = 5, n represents number of biological replicates) and JHH-7 cells (lower, *n* = 3, n represents number of biological replicates). (G) Mitochondrial membrane potential analysis using JC-1 staining following MRPL37 knockdown in SNU-398 and JHH-7 cells (scale bars represent 100 μm). (H) Quantification of the ratio of JC-1 monomers to aggregates in SNU-398 and JHH-7 cells in MRPL37 knockdown cells (*n* = 3, n represents number of biological replicates). Data in D, F, and H are presented as the mean ± SD. ∗*p* < 0.05, ∗∗*p* < 0.01. ∗∗∗*p* < 0.001. ns. Two-tailed Student’s *t* test.

To further explore the effect of MRPL37 on energy metabolism in HCC cells, we performed targeted metabolomics analysis in SNU-398 cells following MRPL37 knockdown. A total of over 60 energy metabolites were detected, of which 16 were upregulated and 6 were downregulated. Notably, the downregulated metabolites included key mitochondrial metabolic products such as ATP, acetyl-CoA, and NAD+ (Figures 7A and 7B). These findings suggest that MRPL37 knockdown significantly impacts mitochondrial metabolism. Differential abundance analysis revealed that MRPL37 knockdown led to metabolic enrichment in pathways associated with glycolysis/gluconeogenesis, carbon metabolism, and the TCA cycle (Figure 7C). ATP assays further demonstrated that the ATP content in MRPL37 knockdown cells was significantly decreased (Figure 7D).

To further investigate the impact of MRPL37 on mitochondrial function, we employed the Seahorse analyzer to assess OXPHOS and glycolysis. The results showed that MRPL37 knockdown significantly inhibited several key aspects of OXPHOS, including basal respiration, maximal respiration, ATP production, and spare respiratory capacity, indicating a profound inhibition of the OXPHOS pathway (Figures 7E and 7F). In contrast, the effects on glycolysis were minimal. Specifically, there were no significant changes in ECAR, which reflects glycolytic activity, including glycolysis rate, maximal glycolytic capacity, and glycolytic reserve (Figures S5A and S5B). Moreover, MRPL37 knockdown led to a significant reduction in mitochondrial membrane potential. JC-1 staining results revealed that the ratio of JC-1 aggregates to monomers was significantly decreased in the shMRPL37 treatment group (Figures 7G and 7H), indicating a decline in mitochondrial membrane potential. These findings collectively suggest that MRPL37 is essential for maintaining mitochondrial function and energy metabolism in liver cancer cells, particularly in regulating OXPHOS.

## Discussion

HCC remains one of the most challenging global health issues, with a high incidence, poor prognosis, and resistance to conventional therapies.^18^^,^^19^ In this study, we constructed a subtype classification and prognostic model for liver cancer based on the MRPL gene family, identifying MRPL37 as a key gene associated with HCC progression. Through the combination of *in vitro* and *in vivo* experiments, along with RNA-seq, proteomics, and targeted metabolomics, we uncovered that MRPL37 may regulate mitochondrial energy metabolism and mitochondrial function, thereby influencing the malignant progression of liver cancer. Our findings suggest that MRPL37 could serve as a therapeutic target in liver cancer.

MRPLs are essential for mitochondrial function, particularly for protein synthesis involved in OXPHOS, which is crucial for cellular energy production. Dysregulation of MRPLs can impact cell survival, proliferation, and metastatic potential in several cancer types, including breast, ovarian, lung, and liver cancers.^12^^,^^20^^,^^21^ This supports MRPLs as potential therapeutic targets for liver cancer. We developed a binary risk model based on MRPL family gene expression, and our model effectively predicted poor prognosis in high-risk groups. The model was associated with key cellular processes such as cell division, cell cycle, and ribosome activity, and also correlated with increased tumor stemness, immune evasion, and resistance to chemotherapy. These results not only highlight the value of our model but also underscore the critical role of MRPLs in liver cancer progression.

The role of MRPL37 in tumor progression has been well-documented across various cancer types, though its function varies between tumors. In lung adenocarcinoma, high expression of MRPL37 correlates with poor prognosis.^22^^,^^23^ However, in colorectal cancer, MRPL37 is downregulated, and its lower expression is associated with increased metastasis.^24^ The differential roles of MRPL37 across various tumors likely reflect differences in tumor stages, metabolic demands, and the tumor microenvironment. In our study, we found that MRPL37 is highly expressed in HCC across multiple tumor databases, and its high expression correlates with poor prognosis in liver cancer patients. Single-cell data, WB, and immunohistochemistry further validated its elevated expression, suggesting its diagnostic and therapeutic value in HCC.

Our analysis also revealed complex interactions between MRPL37 and other MRPL family members. Using LASSO regression, we constructed a prognostic model that incorporates several MRPL family genes, emphasizing the synergistic interactions of MRPL37 with MRPL1, MRPL3, MRPL9, MRPL10, MRPL36, MRPL38, and MRPL52, as well as its antagonistic effects with MRPL46 and MRPL54. Additionally, we have cited recent studies that support these observations. One study found that MRPL37 is associated with metastasis inhibition in colorectal cancer, with interactions between MRPL37 and SLC25A10 through MRPL1 reinforcing its synergistic role.^12^ Conversely, several members of the MRPL family, including MRPL46 and MRPL54, were found to be downregulated in HCC, contributing to malignant progression by impairing OXPHOS.^17^ This finding is consistent with our results and further underscores the antagonistic role of MRPL37 in liver cancer. These findings highlight the unique and critical role of MRPL37 in modulating cancer progression through its interactions with other MRPL family members.

Both univariate and multivariate Cox regression analyses confirmed that MRPL37 has the highest hazard ratio, further establishing it as a critical independent molecular marker for diagnosis and prognosis in liver cancer. Additionally, upregulation of MRPL37 in HCC has been associated with increased tumor stemness, immune evasion, and resistance to immune therapy. Our findings indicate that high MRPL37 expression is significantly associated with the upregulation of immune evasion genes such as PD-L1 and TIM-3, which are known to contribute to immune suppression in the tumor microenvironment. Additionally, TIDE analysis revealed that patients with high MRPL37 expression exhibited higher TIDE scores, suggesting a poorer response to immune checkpoint inhibitors and a greater capacity for immune evasion. These results suggest that MRPL37 may promote immune escape in HCC, potentially through the regulation of immune checkpoint molecules like PD-L1, which inhibit T cell activation and function, thereby evading immune surveillance. Recent studies have highlighted the critical role of mitochondrial ribosomal proteins in regulating the immune microenvironment.^25^ For instance, alterations in MRP expression have been shown to influence the tumor immune microenvironment by modulating immune checkpoint pathways and affecting the metabolic state of immune cells.^26^ These findings underscore the complex interplay between mitochondrial function and immune modulation in cancer progression.

The functional role of MRPL37 in promoting liver cancer progression was further validated through both *in vitro* and *in vivo* models. Silencing MRPL37 in HCC cell lines led to a significant reduction in cell proliferation, cell-cycle arrest at the G0/G1 phase, and induced apoptosis. These findings suggest that MRPL37 is crucial in regulating cell cycle progression and apoptosis in liver cancer cells. Furthermore, MRPL37 knockdown in xenograft and spontaneous liver cancer models significantly inhibited tumor growth, highlighting its essential role in liver cancer tumorigenesis. These data suggest that MRPL37 is vital for maintaining liver cancer survival and proliferation, which makes it a promising target for therapeutic intervention.

Energy metabolism and mitochondrial function are integral to cancer cell survival and proliferation.^27^^,^^28^^,^^29^ Our study highlights the importance of energy metabolism reprogramming in HCC. Dysregulation of mitochondrial protein synthesis and OXPHOS pathways upon MRPL37 knockdown led to a significant reduction in ATP production in HCC cells. This is consistent with the concept that cancer cells undergo metabolic reprogramming to meet the increased energy demands of rapid growth and survival.^30^^,^^31^ Mitochondria are central regulators of cellular energy production and metabolic flexibility. In our experiments, MRPL37 knockdown reduced ATP levels, OXPHOS activity, and mitochondrial membrane potential. These changes triggered compensatory shifts in energy metabolism, including alterations in glycolysis and carbon metabolism. These metabolic shifts underscore the complex internal regulation within the cell in response to MRPL37 depletion. Furthermore, RNA-seq and proteomic data, along with qPCR and WB, revealed that MRPL37 knockdown affected the protein levels of six key mitochondrial energy metabolism proteins—MT-CO1, MT-ND2, MT-ATP6, MT-ATP8, MT-CYB, and MT-CO2—without altering their mRNA levels. This suggests that MRPL37 may play a direct role in mitochondrial protein synthesis at the translational level. Our study demonstrates that MRPL37 plays a pivotal role in the metabolic shift of liver cancer cells by regulating mitochondrial protein synthesis and OXPHOS.

In summary, our study provides strong evidence that MRPL37 plays a pivotal role in regulating mitochondrial function, energy metabolism, and tumor progression in HCC. By influencing key mitochondrial processes, MRPL37 promotes liver cancer progression and contributes to poor prognosis. Our findings suggest that MRPL37 could serve as a potential therapeutic target for liver cancer, offering a foundation for the development of treatment strategies aimed at disrupting mitochondrial function and reprogramming tumor metabolism. Further studies are needed to fully understand the underlying mechanisms and to translate these findings into clinical applications.

### Limitations of the study

While our study provides strong evidence for the role of MRPL37 in liver cancer, several limitations must be acknowledged. First, although we identified MRPL37 as a key gene in HCC, more research is required to further elucidate the molecular mechanisms through which MRPL37 affects mitochondrial function and metabolic pathways. Second, although we have highlighted the clinical relevance of MRPL37 in liver cancer prognosis, additional validation in larger, independent patient cohorts is needed to confirm its potential as a reliable prognostic biomarker.

Additionally, given that the *in vivo* spontaneous tumor model used in this study, AKT/NRAS, induces combined hepatocellular-cholangiocarcinoma, further investigation is required to assess the role of MRPL37 in cholangiocarcinoma cells. Future research should also focus on the biological function of MRPL37 in this context, including the use of a more specific spontaneous hepatocarcinogenesis model (e.g., NRAS/β-catenin).

Furthermore, while our findings suggest that MRPL37 regulates mitochondrial protein synthesis, the direct impact of MRPL37 on the translation efficiency of specific mRNAs remains unclear. To address this, studies such as ribosome profiling are needed to determine whether MRPL37 specifically affects the translation of mitochondrial mRNAs. Finally, the potential therapeutic targeting of MRPL37 in clinical settings will require extensive preclinical and clinical trials to evaluate its safety and efficacy.

## Resource availability

### Lead contact

Further information and requests for resources and reagents should be directed to and will be fulfilled by the lead contact, Zhongji Meng (zhongji.meng@163.com).

### Materials availability

This study did not generate new unique reagents and all materials in this study are commercially available.

### Data and code availability

• The data reported in this paper are available in deposited data in the key resources table.
• This paper does not report original code.
• Any additional information required to reanalyze the data reported in this paper is available from the lead contact upon request.

## Acknowledgments

This work was supported by the 10.13039/100014718National Natural Science Foundation of China (grant no. 82403242, 82573928, Y.Z., Z.M.), and the 10.13039/100017369Scientific and Technological Program of Hubei Province (projects no. 2025AFD228, 2023BCB129, 2024BCB060, 2025DJA043, 2025CCB015, Y.Z., Z.M., D.D., and Z.Y.). 10.13039/501100018773Open Project of Key Project of Hubei Provincial 10.13039/501100016321Clinical Medical Research Center for Precision Diagnosis and Treatment of Liver Cancer (project no. 2024LCOF01, Y.D.).

## Author contributions

Y.Z.: writing – original draft, visualization, resources, project administration, formal analysis, data curation, supervision, funding acquisition, and conceptualization. M.C.: writing – review & editing and data curation. H.L.: data curation and conceptualization. H.D.: data curation and analysis. S.C.: data curation. J.N.: data curation. J.H.: data curation. S.L.: data curation and conceptualization. L.H.: data curation. S.D.: data curation. Z.Y.: data curation. W.Z.: writing – review & editing. D.D.: writing – review & editing. Y.D.: writing – review & editing, data curation, funding acquisition, and conceptualization. Z.M.: writing – review & editing, writing - original draft, funding acquisition, supervision, and conceptualization.

## Declaration of interests

The authors declare no competing interests.

## STAR★Methods

### Key resources table

**Table:** 

REAGENT or RESOURCE	SOURCE	IDENTIFIER
**Antibodies**		

Rabbit polyclonal anti-MRPL37	Proteintech	Cat# 29522-1-AP; RRID:AB_2918321
Rabbit monoclonal anti-β-actin	Servicebio	Cat# GB15003; RRID:AB_3083699
Rabbit polyclonal anti-MTCO1	Abcam	Cat# ab14705; RRID:AB_2084810
Rabbit polyclonal anti-MTND2	Proteintech	Cat# 19704-1-AP; RRID:AB_10638920
Rabbit polyclonal anti-MTATP6	Abcam	Cat# ab190287; RRID:AB_2747745
Rabbit polyclonal anti-MTATP8	Proteintech	Cat# 26723-1-AP; RRID:AB_2880614
Rabbit polyclonal anti-MTCYB	Proteintech	Cat# 55090-1-AP; RRID:AB_2881266
Rabbit polyclonal anti-MTCO2	Abcam	Cat# ab79393; RRID:AB_1603751
Rabbit polyclonal anti-Puromycin	ABclonal	Cat# A23031; RRID:AB_3712837
Rabbit polyclonal anti-Ki67	Abcam	Cat# ab15580; RRID:AB_443209
Recombinant Anti-Cytokeratin 19 antibody (Mouse mAb)	Servicebio	Cat# GB15198; RRID:AB_3712307
Goat polyclonal anti-mouse IgG (H + L) Secondary Antibody, HRP	Thermo Fisher Scientific	Cat# 31430; RRID:AB_228307
Goat polyclonal anti-rabbit IgG (H + L) Secondary Antibody, HRP	Thermo Fisher Scientific	Cat# 31460; RRID:AB_228341

**Chemicals, peptides, recombinant proteins and reagent**		

Protease inhibitor cocktail	Sigma	Cat# P8340
Trizol reagent	Invitrogen	Cat# 15596018
Matrigel	BD	Cat# 356234
Lipofectamine 2000	Invitrogen	Cat# 1668019
PEI 40K	Servicebio	Cat# G1802
Prodidium Iodide (PI) Staining Solution	BD	Cat# 556463
Crystal Violet Staining Solution	Beyotime	Cat# C0121
DMEM/F-12 medium	BDBIO	Cat# L104
RPMI-1640 medium	BDBIO	Cat# L103
DMEM medium	BDBIO	Cat# ST002
Trypsin	BDBIO	Cat# A300
Fetal Bovine Serum (Australian Origin)	BDBIO	Cat# F814

**Critical commercial assays**		

Cell Counting Kit-8	Beyotime	Cat# G1700
Cell Cycle and Apoptosis Analysis Kit	Servicebio	Cat# C1052
Annexin V-FITC/PI Cell Apoptosis Detection Kit	Servicebio	Cat# G1511
Seahorse XF Glycolysis Rate Assay Kit	Agilent	Cat# 103344
JC-1 Mitochondrial Membrane Potential Detection Kit	Servicebio	Cat# G1515
Immunohistochemistry kit	Servicebio	Cat# G1215
Reverse Transcriptase II	Servicebio	Cat# G3416
Fast RNA-seq Lib Prep Kit V2	Aibotech	Cat# RK20306

**Deposited data**		

RNA-seq and data	This paper	https://www.ncbi.nlm.nih.gov/geo/query/acc.cgi?acc=GSE300565
Proteomics data	This paper	https://ngdc.cncb.ac.cn/omix/release/OMIX012335
Metabolomics data	This paper	https://ngdc.cncb.ac.cn/omix/release/OMIX012317
TCGA database	Open-source	https://portal.gdc.cancer.gov/
GEO database	Open-source	http://www.ncbi.nlm.nih.gov/geo/
ICGC database	Open-source	https://dcc.icgc.org/
HPA database	Open-source	https://www.proteinatlas.org/
MRPL family genes	This paper	Table S1

**Experimental models: Cell lines**		

HEK293T cell line	BDBIO	Cat# C5004
SUN-398 cell line	BDBIO	Cat# C5426
JHH-7 cell line	BDBIO	Cat# C5217
Li-7 cell line	BDBIO	Cat# CP-H148
Huh-7 cell line	BDBIO	Cat# C5176
SNU-449 cell line	BDBIO	Cat# C5818
SK-HEP-1 cell line	BDBIO	Cat# C5150
HepG2 cell line	BDBIO	Cat# C5175

**Experimental models: Organisms/strains**		

C57BL/6 mice	Jiangsu Huaxin Innovation Pharmaceutical Technology Co., Ltd	N/A
BALB/c nude mice	Jiangsu Huaxin Innovation Pharmaceutical Technology Co., Ltd	N/A

**Oligonucleotides**		

MRPL37 Forward: TCCCCTGGATAGGGTGTACG	Sangon Biotech	N/A
MRPL37 Reverse: GAGCGGTAGAACCTTGGGT	Sangon Biotech	N/A
ACTB Forward: GGCCAACCGCGAGAAGATGAC	Sangon Biotech	N/A
ACTB Reverse: GGATAGCACAGCCTGGATAGCAAC	Sangon Biotech	N/A
MTCO1 Forward: AAGCCTCCTTATTCGAGCCG	Sangon Biotech	N/A
MTCO1 Reverse: AGAATGGGGTCTCCTCCTCC	Sangon Biotech	N/A
MTND2 Forward: CTTCTGAGTCCCAGAGGTTACC	Sangon Biotech	N/A
MTND2 Reverse: GAGAGTGAGGAGAAGGCTTACG	Sangon Biotech	N/A
MTATP6 Forward: GCTTCATTCATTGCCCCCAC	Sangon Biotech	N/A
MTATP6 Reverse: GATATTGCTAGGGTGGCGCT	Sangon Biotech	N/A
MTATP8 Forward: TGCCCCAACTAAATACTACCGT	Sangon Biotech	N/A
MTATP8 Reverse: AGGATTGTGGGGGCAATGAA	Sangon Biotech	N/A
MTCYB Forward: AACTTCGGCTCACTCCTTGG	Sangon Biotech	N/A
MTCYB Reverse: TGATCCCGTTTCGTGCAAGA	Sangon Biotech	N/A
MTCO2 Forward: TGCAGCGCAAGTAGGTCTAC	Sangon Biotech	N/A
MTCO2 Reverse: AAGCCTAATGTGGGGACAGC	Sangon Biotech	N/A
shRNA targeting sequence: Ctrl: CAACAAGATGAAGAGCACCAA	Tsingke Biotech	N/A
shRNA targeting sequence: MRPL37-1#: GCTCACCAAGACCAAGTTAAT	Tsingke Biotech	N/A
shRNA targeting sequence: MRPL37-2#: GAAGCTACTAAGAATCATGTT	Tsingke Biotech	N/A
shRNA targeting sequence: MRPL37-3#: CCAGCTCCTCTATCAGCATTT	Tsingke Biotech	N/A
siRNA targeting sequence:MRPL37-944-s: CCCUGUACUUACUGGACAATT	Sangon Biotech	N/A
siRNA targeting sequence:MRPL37-944-a: UUGUCCAGUAAGUACAGGGTT	Sangon Biotech	N/A

**Recombinant DNA**		

pLKO.1-shCtrl	This paper	N/A
pLKO.1-shMRPL37-puro	This paper	N/A
pcDNA3-FLAG-tagged hMRPL37	This paper	N/A

**Software and algorithms**		

ImageJ	NIH	https://imagej.nih.gov/ij/
R Studio	The R foundation	https://www.r-project.org/
Adobe Illustrator	Adobe	https://www.adobe.com/
GraphPad Prism 8	GraphPad Sofware	https://www.graphpad.com/

**Other**		

Laser scanning confocal focus microscope	NOVEL	NCF-1000
Flow cytometer	Challenbio	FongCyte™3
IHC h-score analysis	PathologyProfiler	V0.1.4

### Experimental model and study participant details

#### Clinical sample

This study included surgical specimens from 28 HCC patients. All procedures were approved by the Ethics Committee of Hubei University of Medicine (2025-PR-172), and written informed consent was obtained from each participant. The study adhered to the ethical principles outlined in the Declaration of Helsinki. Tumor tissues and matched adjacent non-tumor liver tissues, designated as experimental and control groups, were obtained from HCC patients who had not received prior systemic anti-cancer therapy.

#### Cell culture

The HCC cell lines SNU-398 and JHH-7, and HEK-293T cells were cultured in Dulbecco's modified Eagle's medium (DMEM) supplemented with 10% fetal bovine serum and 1% penicillin-streptomycin at 37°C in 5% CO2. All cell lines were authenticated using short tandem repeat analysis and confirmed to be free of mycoplasma contamination by PCR testing.

#### Mice

6-week-old male C57BL/6 and BALB/c nude mice were obtained from Jiangsu Huaxin Innovation Pharmaceutical Technology Co., Ltd. All mice were housed in a specific pathogen-free facility at Hubei University of Medicine. All animal experiments were approved by Ethics Committee of Hubei University of Medicine.

### Method details

#### Consensus clustering analysis of MRPLs

Consensus clustering was performed using the R package “ConsensusClusterPlus (v1.54.0)”.^32^ The clustering criteria included a relatively flat cumulative distribution function (CDF) curve, ensuring balanced sample sizes for each subtype and increased within-subtype correlation with decreased between-subtype correlation. Based on the expression profiles of prognostic MRPLs, samples were classified into distinct risk subtypes. Cluster heatmap analysis was conducted using the “pheatmap” R package (v1.0.12), including only genes with a variance greater than 0.1. The top 25% of genes with the highest variance were extracted and displayed. Principal Component Analysis (PCA) was performed using the “ggplot2” R package to observe the distinction between the risk subgroups.

#### Construct the risk score model based on MRPLs

To construct an MRPL gene signature, we first analyzed the TCGA HCC cohort to identify genes associated with patient prognosis. Lasso Cox regression analysis was performed using the R package “glmnet” based on differentially expressed prognostic genes. The optimal lambda value (λ) for the LASSO model was selected through 10-fold cross-validation, aiming to minimize the mean cross-validation error. This process was executed using the cv.glmnet () function in the “glmnet” package, which identifies the lambda value that minimizes the cross-validation error (lambda.min). Based on this analysis, we constructed a risk score model by evaluating the trajectory of each gene. The formula for the risk score is as follows: Risk score = Σ (Coefi × Exp), where Coefi represents the risk coefficient and Exp represents the gene expression level. Based on the median risk score, samples were categorized into high-risk and low-risk groups. Kaplan-Meier survival analysis was conducted to compare overall survival (OS) between the high-risk and low-risk groups. Receiver Operating Characteristic (ROC) curves were constructed to assess the prognostic prediction performance of the model.

#### Plasmid construction and transfection

Lentivirus packaging was performed using PEI 40K Transfection Reagent to transfect HEK-293T cells with psPAX2, pMD2.G, and shRNA-containing plasmids. Supernatants were collected 48 hours post-transfection, and target cells were infected for 48 hours. Stable cell lines were selected using 1 μg/mL puromycin.

#### Cell proliferation and colony formation assays

Cell proliferation was assessed using the CCK-8 assay. Cells were seeded in 96-well plates, and CCK-8 solution was added at 0, 24, 48, 72, and 96 hours. Absorbance at 450 nm was measured after 3 hours of incubation. For colony formation assays, cells were seeded in 6-well plates and incubated for 7–14 days. Colonies were fixed, stained, and counted using Image J software.

#### Cell cycle and apoptosis analysis

For cell cycle analysis, cells were fixed in 75% ethanol overnight at -20°C, stained with PI for 30 minutes, and analyzed using flow cytometry. For apoptosis detection, cells were stained with Annexin V-FITC and PI, and the apoptotic cell population was measured by flow cytometry.

#### Animal model

For the AKT-NRAS-induced liver cancer mouse model, 6-week-old male C57BL/6 mice were injected via tail vein with the plasmid mixture (pT3EF1a-AKT-HA, 2 μg; pcAG-NRAS, 8 μg; SB100, 1 μg) to induce tumor formation. Starting at week 9, shMRPL37-AAV was administered *via* tail vein to silence MRPL37, and euthanasia was performed at week 15. Liver tissues were harvested for gross imaging analysis, and tumor size and number were quantitatively assessed. Serum samples were collected for AST and ALT level measurements, and histopathological analysis was performed. For the immunodeficient mouse liver cancer model, BALB/c-nu nude mice were subcutaneously injected with SNU-398 cells (1×10^7^ cells/100 μL) and tumor volume was monitored. Tumor volume was calculated using the formula V = 0.52 × L × W^2^, where L represents the longest diameter and W the perpendicular diameter.

#### Immunohistochemistry staining

Tumor tissue slides from mice were incubated with Ki67 and CK-19 antibodies. Visualization was achieved through diaminobenzidine treatment followed by hematoxylin counterstaining. Whole-slide images were acquired using a GScan-20 digital scanner, and H-score quantification was performed using Pathology Profiler software (version 0.1.4).

#### Western blot analysis

Western blot analysis was performed as previously described.^33^ Briefly, HCC cells were lysed using RIPA lysis buffer supplemented with a 1 × protease inhibitor cocktail. Protein samples were separated by SDS-PAGE, transferred to PVDF membranes. The membranes were blocked with 5% skimmed milk and then incubated overnight at 4°C with primary antibodies. Following primary antibody incubation, the membranes were incubated with the secondary antibody at room temperature for 1 hour. Protein bands were visualized using the ECL detection reagent.

#### RT-qPCR

Total RNA was extracted using TRIzol reagent and quantified using a NanoDrop spectrophotometer. cDNA synthesis was performed using Reverse Transcriptase II. qPCR was performed using Universal Blue SYBR Green Master Mix on an ABI StepOne Plus system. PCR conditions included an initial denaturation at 95°C for 3 minutes, followed by 40 cycles of 95°C for 30 seconds, 60°C for 30 seconds, and 72°C for 30 seconds. Data were analyzed using the 2^−ΔΔCt^ method, with ACTB as the reference genes.

#### ECAR and OCR measurements

The extracellular acidification rate (ECAR) and oxygen consumption rate (OCR) were measured using the Seahorse XFe96 analyzer. Cells were incubated with XF assay medium and subjected to the Seahorse XF Cell Mito Stress Test and Glycolysis Stress Test. These tests were performed with the appropriate substrates and inhibitors, including Oligomycin, FCCP, Rotenone/antimycin A (ROT-AA), glucose, and 2-deoxy-D-glucose (2-DG). The data were analyzed to evaluate mitochondrial function and glycolytic capacity.

#### Mitochondrial membrane potential detection

Mitochondrial membrane potential was assessed using JC-1 detection kits. Cells were incubated with JC-1 dye for 30 minutes. The nuclei were stained with Hoechst 33342. After incubation, slides were mounted and observed using a laser scanning confocal microscope. Fluorescence intensity was measured to quantify changes in mitochondrial membrane potential.

#### RNA-seq analysis

Total RNA was extracted from cells using TRIzol reagent and purified through phenol–chloroform extraction following the manufacturer’s instructions. RNA quality and integrity were assessed using an Agilent 2100 Bioanalyzer. Ribosomal RNA was depleted using the Ribo-Zero kit, and mRNA was enriched and fragmented for library construction using the Fast RNA-seq Lib Prep Kit V2. The sequencing libraries were quantified by qPCR and size-validated by Bioanalyzer, then subjected to paired-end 150 bp (PE150) sequencing on an Illumina NovaSeq 6000 platform. Raw reads were quality-controlled using FastQC (v0.11.9) and trimmed with Trimmomatic (v0.39) to remove low-quality bases and adapters. Clean reads were aligned to the homo sapiens reference genome (GRCh38) using HISAT2 (v2.2.1). Gene expression levels were quantified as fragments per kilobase of exon per million reads (FPKM) using featureCounts (v2.0.1). Differentially expressed genes were identified with DESeq2 (v1.36.0) in R (v4.2.0) using adjusted p < 0.05 as the significance cutoff. Functional enrichment analyses (GO and KEGG) were performed with ClusterProfiler (v4.2.2).

#### Mass spectrometry analysis

Protein samples were extracted and sonicated in urea buffer. After protein concentration determination, digestion was performed using trypsin, and peptides were desalted using C18 columns. LC-MS/MS was used to analyze peptide sequences and identify proteins.

#### Metabolic mass spectrometry analysis

Cell pellets were washed with ice-cold PBS, resuspended in ultrapure water, and extracted with 80% methanol to precipitate proteins. The mixture was vortexed, sonicated, and centrifuged. The supernatant was dried under nitrogen and reconstituted in 50% methanol for LC-MS analysis. Metabolomic profiling was conducted using a Waters ACQUITY UPLC H-Class system coupled to a QTRAP® 6500+ mass spectrometer equipped with an electrospray ionization (ESI) source operating in both positive and negative ion modes. Chromatographic separation was achieved on a Waters ACQUITY UPLC BEH C18 column at 40 °C with mobile phases consisting of 0.1% formic acid in water and 0.1% formic acid in acetonitrile. Data acquisition and peak integration were performed using Analyst 1.6.3 software. Feature detection, alignment, and normalization were carried out using XCMS (v3.16.1) in R. Metabolite identification was confirmed by matching retention times and MS/MS spectra with entries in the HMDB and METLIN databases. Multivariate statistical analyses, including PCA and PLS-DA, were performed with MetaboAnalyst 5.0.

### Quantification and statistical analysis

All statistical analyses were processed on R Studio or GraphPad Prism 8 software. Statistical analyses were performed using Student’s two-tailed t-test or one-way ANOVA followed by Tukey's post-test. For time-dependent data, two-way ANOVA with post-hoc tests was applied. Survival curves were generated with the Kaplan-Meier method and log-rank tests. Cox regression analysis was conducted using SPSS 19.0. Quantitative results are expressed as the mean ± standard deviation (SD). A p value of less than 0.05 was considered statistically significant. All of the statistical details of experiments, including the sample number and the statistical tests used have been listed in the figures and figure legends.
