# Supplementary material for: MRPL37 promotes hepatocellular carcinoma progression through modulating mitochondrial energy metabolism
Source: iScience. 2025 Nov 14;28(12):114052. doi: 10.1016/j.isci.2025.114052 (PMC12702225; doi:10.1016/j.isci.2025.114052)
Supplement: Document S1. Figures S1–S5 and Tables S1 and S2 [file mmc1.pdf]

iScience, Volume *28*

Supplemental information

MRPL37 promotes hepatocellular carcinoma progression through modulating mitochondrial energy metabolism

Yigan Zhang, Minjie Chen, Huidi Li, Hao Deng, Shuwen Chen, Jiaxin Ni, Junjie Hu, Sixian Lei, Linsheng Huang, Shuangsuo Dang, Zhuoshun Yang, Wuhua Zhou, Deping Ding, Yanbin Dong, and Zhongji Meng

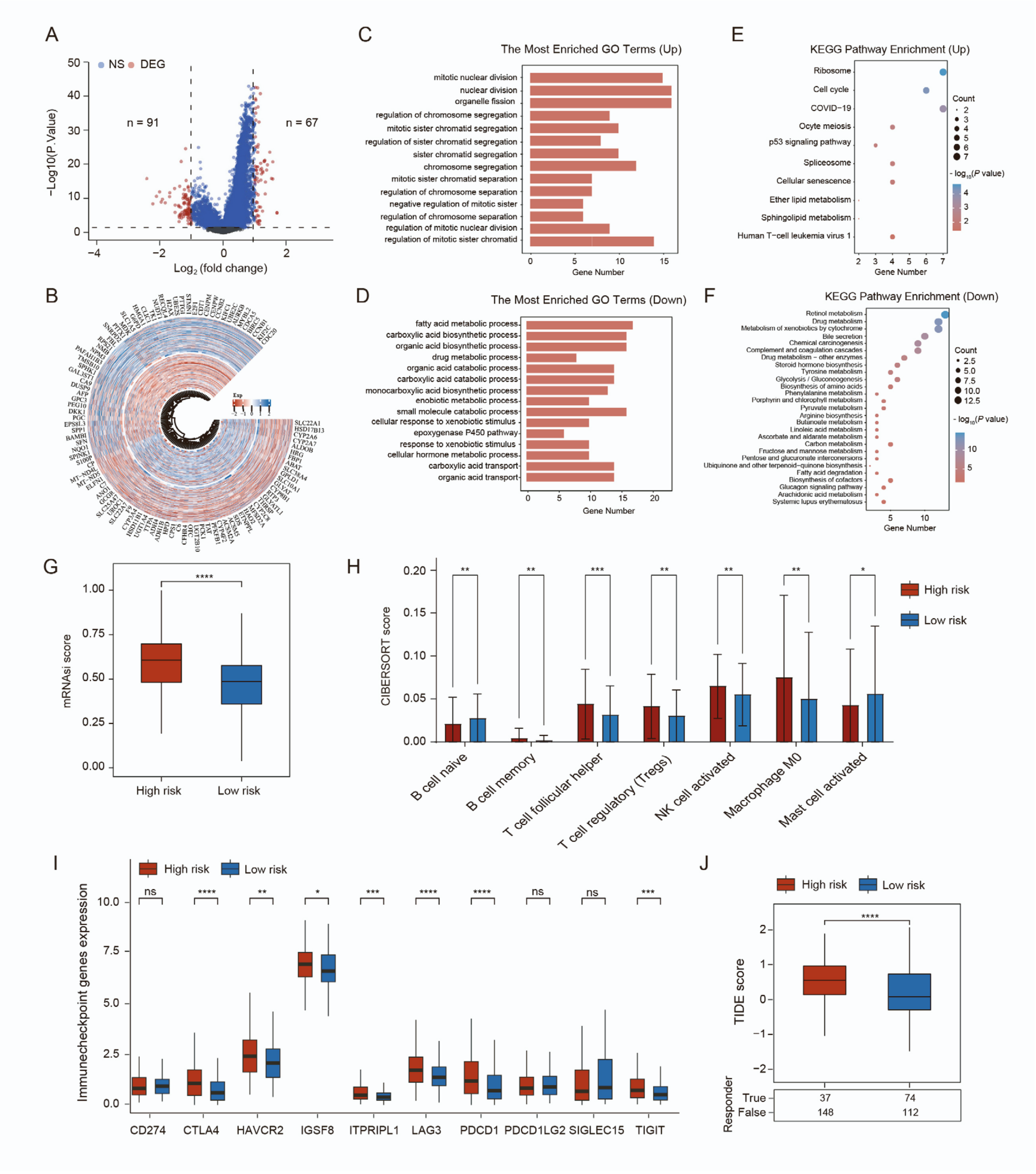

Figure S1. Functional enrichment, tumor microenvironment, and immune therapy characteristics of MRPL subtypes.

(A-B) Volcano plot and heatmap showing differential gene expression based on MRPL subtypes in HCC from the TCGA dataset. (C-D) GO and KEGG enrichment analyses highlighting the biological pathways significantly associated with MRPL subtypes. (E) Comparison of mRNA stemness index (mRNAsi) scores between high-risk and low-risk MRPL subtypes. (F) Distribution of immune cell infiltration across MRPL subtypes as determined by CIBERSORT analysis. (G) Comparison of immune checkpoint gene expression between high-risk and low-risk MRPL subtypes. (H) Comparison of TIDE

scores between high-risk and low-risk MRPL subtypes.

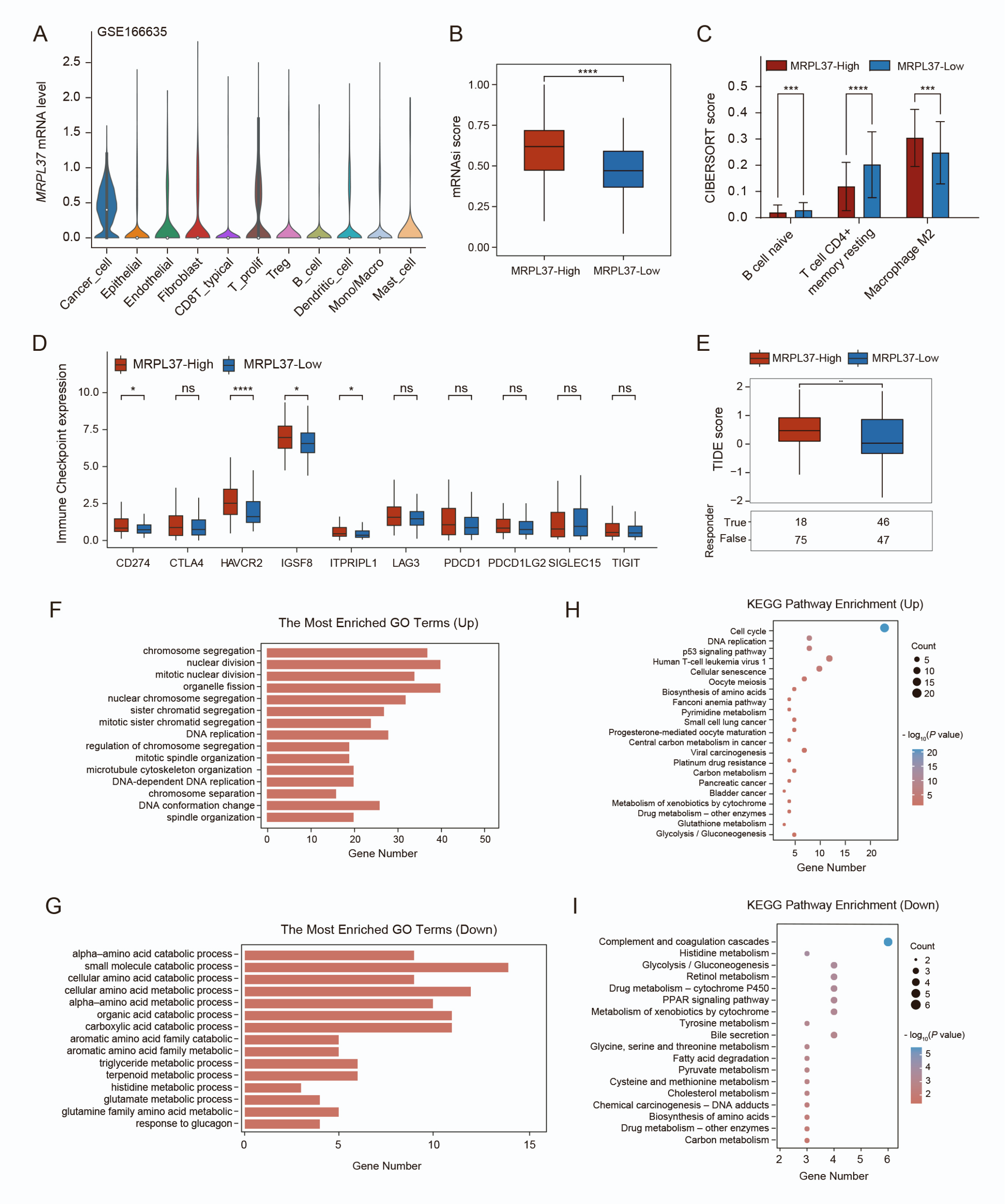

Figure S2. Functional enrichment, tumor microenvironment, and immune therapy characteristics associated with MRPL37 expression.

(A) Violin plot showing MRPL37 mRNA expression across different cell types in the GSE166635 dataset. (B) Analysis of mRNAsi scores between MRPL37-high and MRPL37-low groups. (C) CIBERSORT scores showing the distribution of immune cell types between MRPL37-high and MRPL37-low groups. (D) Expression levels of immune checkpoint genes in MRPL37-high and MRPL37-low groups (E) Comparison

of TIDE scores between MRPL37-high and MRPL37-low groups, with MRPL37-high showing a higher responder rate. (F-G) GO enrichment analysis identifying the enriched GO terms associated with MRPL37 expression. (H-I) KEGG pathway enrichment analysis showing the most significant pathways associated with MRPL37 expression.

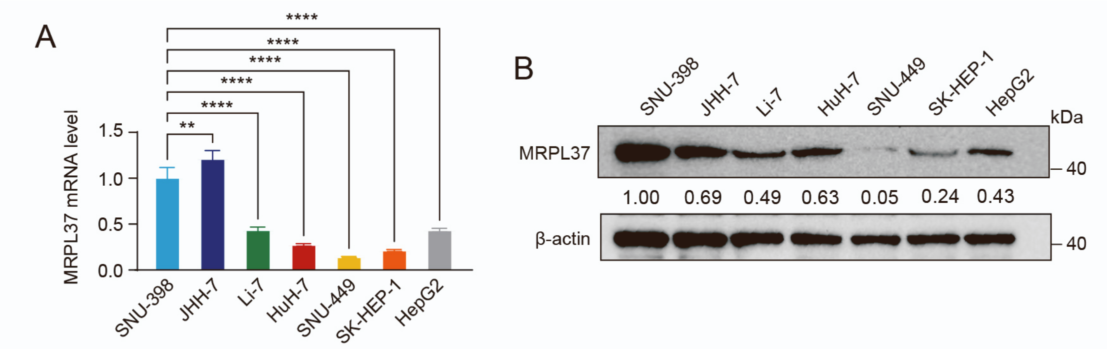

Figure S3. Expression levels of MRPL37 in HCC cell lines.

(A) qPCR analysis showing relative MRPL37 mRNA expression levels in different HCC cell lines. (B) WB analysis confirming MRPL37 protein expression levels in different HCC cell line. Data in A are presented as the mean ± SD (n = 3, n represents number of biological replicates). **p < 0.01. **** p < 0.0001. One-way ANOVA with Tukey's test.

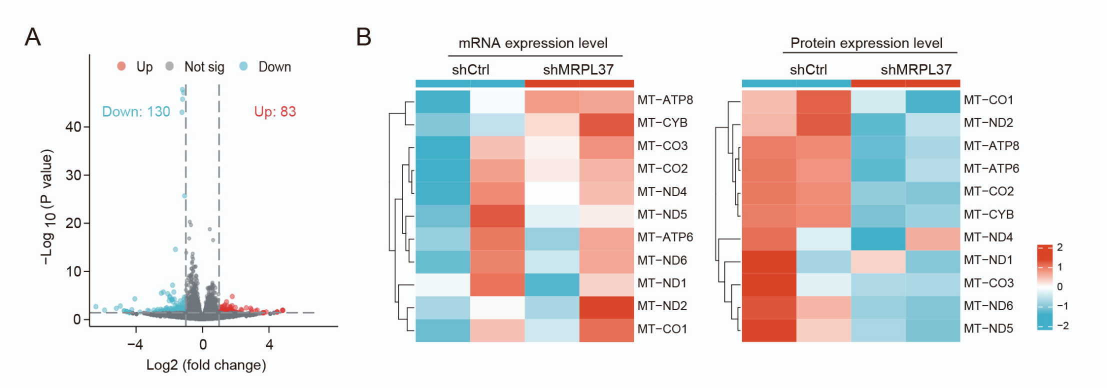

Figure S4. Transcriptomic analysis following MRPL37 knockdown in SNU-398 cells.

(A) Volcano plot displaying differentially expressed genes (DEGs) between the shCtrl and shMRPL37 groups. (B) Heatmap analysis of mRNA and protein levels of mitochondrial genes (MT-CO1, MT-ND2, MT-ATP6, MT-ATP8, MT-CYB, MT-CO2) in SNU-398 cells following MRPL37 knockdown.

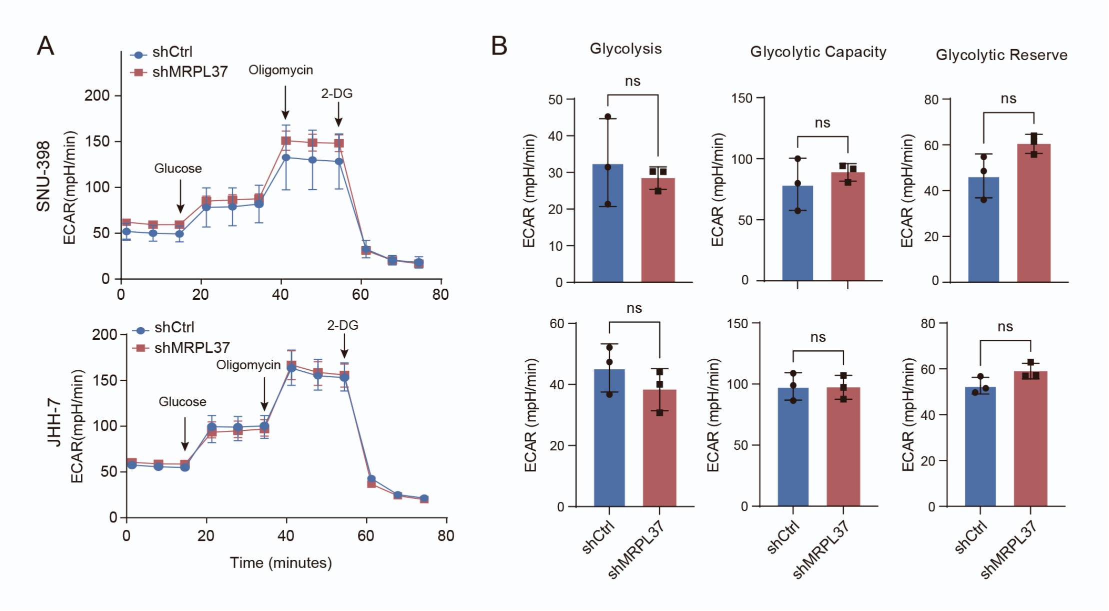

Figure S5. Analysis of extracellular acidification rate (ECAR) in SNU-398 and JHH-7 cells following MRPL37 knockdown.

(A) ECAR measurements over time in SNU-398 and JHH-7 cells following MRPL37 knockdown or control treatment. (B) Quantification of glycolysis (left), glycolytic capacity (middle), and glycolytic reserve (right) based on ECAR measurements in SNU-398 (upper) and JHH-7 cells (lower). Data in B are presented as the mean ± SD (n = 3, n represents number of biological replicates). ns., not significant. Two-tailed student's t-test.

Supplementary Table 1. MRPL family genes

**MRPL family genes**
MRPL1
MRPL2
MRPL3
MRPL4
MRPL9
MRPL10
MRPL11
MRPL12
MRPL13
MRPL14
MRPL15
MRPL16
MRPL17
MRPL18
MRPL19
MRPL20
MRPL21
MRPL22
MRPL23
MRPL24
MRPL27
MRPL28
MRPL30
MRPL32
MRPL33
MRPL34
MRPL35
MRPL36
MRPL37
MRPL38
MRPL39
MRPL40
MRPL41
MRPL42
MRPL43
MRPL44
MRPL45
MRPL46
MRPL47
MRPL48
MRPL49

MRPL50
MRPL51
MRPL52
MRPL54
MRPL55

Supplementary Table 2. Correlation between MRPL37 expression and clinical pathological characteristics in HCC

Characteristics	Low expression of MRPL37	High expression of MRPL37	P value
n	187	187	
Age, n (%)			0.878
<= 60	89 (23.9%)	88 (23.6%)	
> 60	97 (26%)	99 (26.5%)	
Gender, n (%)			0.060
Female	69 (18.4%)	52 (13.9%)	
Male	118 (31.6%)	135 (36.1%)	
Pathologic stage, n (%)			0.099
Stage I	97 (27.7%)	76 (21.7%)	
Stage II	37 (10.6%)	50 (14.3%)	
Stage III&Stage IV	43 (12.3%)	47 (13.4%)	
Pathologic T stage, n (%)			**0.036**
T1	104 (28%)	79 (21.3%)	
T2	40 (10.8%)	55 (14.8%)	
T3&T4	42 (11.3%)	51 (13.7%)	
Pathologic N stage, n (%)			0.614
N0	126 (48.8%)	128 (49.6%)	
N1	3 (1.2%)	1 (0.4%)	
Pathologic M stage, n (%)			0.135
M0	133 (48.9%)	135 (49.6%)	
M1	4 (1.5%)	0 (0%)	
